# Prevalence and factors associated with acute malnutrition among children with epilepsy at the national referral hospital in Uganda, a cross sectional study

**DOI:** 10.21203/rs.3.rs-7543730/v1

**Published:** 2025-09-17

**Authors:** Juliana Kayaga, Nicolette Nabukeera Barungi, Anthony Batte, Richard Idro

**Affiliations:** Makerere University; Makerere University; Makerere University; Makerere University

**Keywords:** Epilepsy, Acute Malnutrition

## Abstract

**Background:**

Sub-Saharan Africa carries a significant burden of epilepsy and malnutrition. Although both epilepsy and malnutrition are widespread in low and middle income countries, the burden of acute malnutrition among children living with epilepsy is still unknown. This study aimed to determine the prevalence and factors associated with acute malnutrition among children with epilepsy.

**Methods:**

This cross-sectional study enrolled children with epilepsy aged 6 months to 12 years attending the paediatric neurology outpatient clinic at Mulago National Referral Hospital, Uganda. Enrolled children underwent clinical exam and clinical history was collected. We used World Health Organization (WHO) growth reference z-scores to categorize children and defined acute malnutrition as children with weight for height z-scores or BMI for age Z score of < −2 Standard deviation. To determine the associated factors, multivariable logistic regression analysis was done and adjusted odd ratios(aOR) as well as 95% confidence interval (CI) was used to estimate the strength of the association. A p-value of

**Results:**

We enrolled 280 children with epilepsy. The median age (IQR) was four years (1.9–7.5) and 187 (66.8%) were male. Out of the 280 participants, 44 (15.7%) had acute malnutrition. The factors associated with increased odds of acute malnutrition included gross motor impairment (aOR 8.33, 95% CI: 2.27–34.50, p = 0.002), ongoing seizures in the last 6 months (aOR 5.65, 95% CI: 1.34–33.90 p = 0.03) and feeding difficulties (aOR 3.19, 95% CI: 1.23–8.54, p = 0.018). On the other hand, school attendance (aOR 0.12, 95% CI: 0.03–0.41, p = 0.001), rural residence (aOR 0.5 95% CI: 0.22–0.99, p = 0.048) and caregiver with primary or no education (aOR 0.4, 95% CI: 0.19–0.88, p = 0.018), were associated with reduced odds of acute malnutrition.

**Conclusion:**

The prevalence of acute malnutrition was high among this vulnerable population of children. There is a need to routinely carry out nutritional assessment for children with epilepsy especially those with ongoing seizures, motor disabilities and feeding disabilities.

## Background

Globally, approximately 70 million people live with epilepsy (PWE) 80% of whom are in low- to middle-income countries (1). The prevalence of epilepsy in Sub-Saharan Africa ranges from 10 to 50 per 1000 children compared to only 5 to 10 per 1000 in the western world (1). In Uganda, almost 2% of the population has active epilepsy (2).

Acute malnutrition affects up to 7% of children world-wide and 4% of all children below 5 years in Uganda(3). It also contributes to 45% of mortalities seen in this age group. Children with epilepsy are at an increased risk of acute malnutrition due to factors that reduce the energy intake or disproportionately increase the energy expenditure (4). Such factors include comorbidities like cerebral palsy, intellectual disability, feeding difficulties and recurrent seizures requiring hospital admissions (4–6). Moreover, social factors like food taboos, social stigma and deprivation may predispose them to acute malnutrition(7). Conversely, the presence of acute malnutrition among this population may complicate the management of epilepsy(8). Biochemical variations that occur in severe acute malnutrition such as hyponatreamia, hypocalcemia, hypoglycaemia and reduction in inhibitory neurotransmitters like gamma aminobutyric acid (GABA) reduce the seizure threshold (4, 9). In addition, malnourished children are highly predisposed to infections and fevers which trigger seizures (3, 4). A community study in rural Benin showed the prevalence of acute malnutrition among children with epilepsy to be 13.8%, which was higher than the population without epilepsy (4, 7, 10). Hemamalini et al in India found 40% of children with drug resistant epilepsy were at high risk of malnutrition (6). However within the east African region this information is scarce and yet epilepsy etiologies and patterns may be different. The burden of acute malnutrition among children with epilepsy in Uganda is currently undocumented and there are no routine nutritional assessments of these children as part of the national guidelines of care for epilepsy. This study aimed to determine the prevalence and factors associated with acute malnutrition among children with epilepsy at Uganda’s national referral hospital.

## Methodology

### Study Design and setting

This was a cross-sectional study carried out from April 2021 to August 2021. It was conducted in the paediatric neurology clinic (PNC) at Mulago National Referral Hospital in Kampala, Uganda. Mulago National Referral hospital is a public hospital located 2 km from the Capital city centre, Kampala and serves as a National Referral for the entire country. It also serves as a teaching hospital for Makerere University College of Health Sciences, Uganda’s biggest and oldest medical school. The paediatric neurology clinic is run as an outpatient specialized clinic which caters to children with neurological disorders, twice a week, every Monday and Thursday between 8 am and 5 pm. It serves as a referral outpatient clinic for neurological cases from all over the country. Annually, the clinic enrols about 300 new patients with neurological illnesses. On each clinic day, approximately 30 to 40 children with epilepsy are attended to however, many are repeat visits. Patients are seen by a team composed of a paediatric neurologist, paediatricians and residents. The team does not include a nutritionist. Children seen in this clinic are referrals from paediatric wards and other hospitals around the country with some self-referrals. Services are offered free of charge apart from some investigations which are paid for at the hospital. Patients are given appointments of 2 weeks up to 3 months based on their condition and follow up requirement. If a patient is found to have malnutrition, they are referred to the out-patient therapeutic centre based at the nutrition unit of the hospital that runs every Wednesday.

### Study Population and sample size

The study enrolled all children with epilepsy aged 6 months to 12 years who attended the neurology clinic at Mulago national referral hospital during. We defined epilepsy according to international league against epilepsy (ILAE) as a neurological disorder in a patient with at least 2 unprovoked (reflex) seizures occurring 24 hours apart or a patient with a documented diagnosis of an epilepsy syndrome. Children that were too sick to withstand study procedures such as those with active convulsions, and those whose parents denied consent were excluded. A total of 282 participants were consecutively selected and only 2 were excluded due to severe sickness that required them to move to the emergency unit of the hospital. All patients approached to join the study accepted.

### Data collection

Data were collected by the principal investigator (PI) and four research assistants: one medical officer and three nurses. The research assistants had a one-day training on the study objectives and protocol. Assent for the children aged eight years and above and informed consent was sought from a parent or caregiver of an eligible child prior to enrolment into the study. For all recruited children, the demographic factors, patient medical history including description of seizures, drugs taken, comorbidities, immunisation, growth and development, dietary history and family social history were recorded on a data collection tool. Feeding difficulties were defined as failure to chew, failure to swallow, poor appetite, food unintentionally falling out of the mouth and distressing meal time.

The patient’s hospital records were reviewed to get data on diagnosis as per clinician, anti-epileptic drugs prescribed and any other diagnosed comorbidities. A physical examination was performed on the child including physical signs commonly seen in acute malnutrition and neurology exam focusing on general observation and motor exam for disability. Anthropometric measurements, weight, length/height and Mid Upper Arm Circumference were taken. To measure the length or height of the child; a measuring board with a headboard and sliding foot piece (Seca 213 Stadiometer) was used. For length, the child was positioned lying on his/her back on the measuring board, supporting the head and placing it against the headboard. The occiput, shoulder blades, gluteus muscle, cuffs and heels had to all touch the board. The length was measured to the last completed 0.1 cm and recorded immediately. This was done for children below 2 years or those shorter 87cm. For those above 2 years, height was measured. The same stadiometer was used with a vertical back board, a fixed base board, and a movable headboard. The stadiometer was be placed on a level floor. The child was barefoot with no hair ornaments or braid buns. The length/height was measured three times and an average obtained which was considered as the correct value. Special consideration was made for children with deformities who were unable to lie straight or stand, tibial length was used to estimate the height of the child. The distance between the medial edge of the Tibia to the inferior medial edge of the Malleolus was measured with the use of a flexible tape. The result was placed into a formula as follows: (3.26 × tibial length) + 30.8cm = length of the child (11).

The weight was measured using a well calibrated 2 in 1 weighing Seca scale. In a private room, all of the child’s clothes were removed including diapers. The scale was adjusted to 0 and the child allowed to stand on the scale. The weight was recorded to the nearest 0.01kg. For infants or children unable to independently stand on the scale due to disability or lack of cooperativeness, the caretaker was requested to stand on the scale, after the weight was measured, the scale was adjusted to 2 in 1 mode. Once it went back to zero, the caretaker was asked to carry the child, without going off the scale. The new weight was recorded as the child’s weight. The measurement was done twice, and the average taken as the correct value. Acute malnutrition or wasting was defined as weight for height (WFH) or weight for length (W/L) or body mass index (BMI) for age z-score less than − 2 standard deviation from the median WHO growth standards (12, 13). Additionally, a blood sample was taken off for a complete blood count and HIV test.

### Data management and analysis

The data collected were checked for completeness and correctness by the principal investigator. The study questionnaires and other data collection instruments were checked to ensure there were no errors. A data base was set up using Epi data version 4.4.1. The data were double entered and the two entry versions checked for consistency. The data were then cleaned, edited and backed up on password protected external drive. Data entered in epi-data software were exported into STATA statistical software version 14 for analysis. Emergency Nutrition Assessment (ENA2020) software was used to obtain weight for height z-scores for participants below 5 years and World Health Organisation AnthroPlus software was used to obtain BMI for age z-scores for those 5 years and above. The continuous variables were summarized using median and interquartile range and categorical variables using frequencies and percentages. To determine the association between the various independent variables in this study and acute malnutrition, logistic regression analysis method was used. At multivariable analysis, associations with p-value < 0.05 were considered to be significant after assessing for interactions between variables followed by assessment of confounding effect of one independent variable on the relationship between another independent variable with acute malnutrition. A percentage difference between crude prevalence ratio and adjusted prevalence ratio of < 10% signified absence of confounding effect.

## Results

We enrolled 280 children with epilepsy. The median age (IQR) was 4.2 (2– 5.8) years and 162 (57.8%) children were below the age of 5 years. Majority of the participants were boys 187 (66.8%) and 197 (70.6%) lived in an urban residence. With regard to the caregivers, majority 245 (88.6%) were parents to the participants and 76 (27.6%) had a highest education level of primary school and below. The youngest caregiver was 19 years and the oldest 65 years. 202 (72%) of the caregivers were aged between 25 and 45 years. The median (IQR) duration of epilepsy was 25.8 months (11.5 – 49.5) but only 42 (15%) had a duration of less than one year. Generalized seizures were the most common 161(57.5%) and 47(16.9%) of the participants had an epilepsy syndrome. Cerebral palsy was the leading comorbidity affecting 103 (36.9%) of the participants. Of the participants, 11(4.0%) were no longer taking anti-epileptic drugs and 123(44.1%) took herbal medications together with the antiepileptic drugs. (Table 1 and Table 2)

### Prevalence of acute malnutrition:

**Figure 1:**
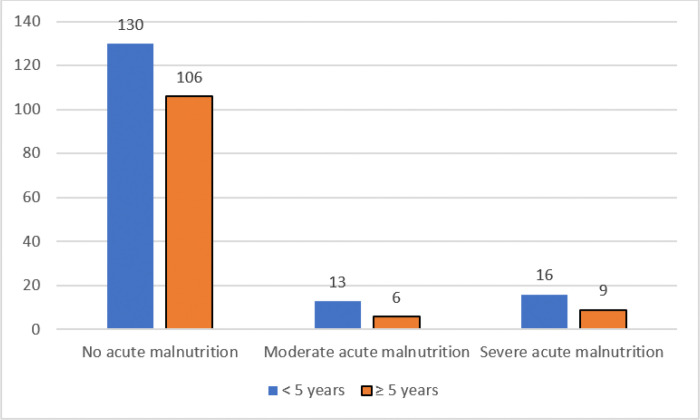
A bar graph showing the distribution of acute malnutrition by age category

Among the 280 participants enrolled into the study, 44 (15.7%) had acute malnutrition. Of the participants with acute malnutrition, 19 (43.2%) had moderate acute malnutrition (MAM), while 25 (56.8) had severe acute malnutrition (SAM). There was no child with edematous malnutrition. Over half of the children 29 (65.9%) were males, 29 (65.9%) were below the age of five years and 35 (81.4%) hailed from an urban residence. The commonest seizure type was generalized seizures 25 (56.8%) whereas 36 (81.8%) had a motor disability involving the limbs. Figure 1 shows the distribution of acute malnutrition by age.

### Factors associated with acute malnutrition.

Children with gross motor impairment were eight times more likely to have acute malnutrition compared to those without (aOR 8.33 95% CI 2.27–34.5 p value 0.002). The odds were also increased among those with feeding difficulties (aOR 3.19, 95% CI, 0.23–8.54, p value 0.018). For children with ongoing seizures over the last 6 months prior to enrolment, the likelihood of acute malnutrition was 5 times more than those who had controlled seizures (aOR 5.65 95% CI 1.34–33.9 p value 0.032). It was however noted that among school going age children, those that were attending school were less likely to have acute malnutrition compared to those that do not attend school (aOR 0.12 95% CI 0.03–0.41 p value 0.001). Additionally, children whose parents had a level of education of primary school or lower also were less likely to have acute malnutrition compared to those with a tertiary education (aOR 0.4, 95% CI 0.187–0.854, p value 0.018). Table 3 shows the factors with adjusted odds ratio.

## Discussion

The study found that the prevalence of acute malnutrition among children with epilepsy was 15.7% which was nearly four times higher that reported by Uganda demographic household survey (UDHS) 2016, where only 4% of Ugandan children under five years had acute malnutrition (14). However, for children with epilepsy, studies have reported a range between 13–52% in agreement with the findings of this study (7, 15, 16). In this study boys made up almost 2 thirds of the children with acute malnutrition. This was not surprising because the study population also had a proportion of 2 thirds being male participants. There were more children with severe acute malnutrition than moderate malnutrition. This could be explained by the significant presence of motor disabilities and feeding difficulties among these children that could severely impede their nutritional intake leading to severe forms of acute malnutrition.

We found that participants with a gross motor impairment were eight times more likely to have acute malnutrition compared to those without this disability. A significant proportion of children with neurological disabilities have been associated with high risk of poor nutritional status, particularly those with severe and longer-term gross motor impairment (17). In particular, cerebral palsy has shown to have high levels of acute malnutrition due to high resting basal metabolic rate. This increases the energy requirement but the demand may not be met especially in a low resource setting (18). Additionally, children with disabilities face difficulties in accessing food by themselves and are prone to social deprivation due to stigma and caregiver fatigue (19, 20). Feeding difficulties were associated with three times increased odds of acute malnutrition. This finding is in tandem with H Tekin et al 2018 who found that among children with epilepsy, the prevalence of malnutrition was higher among those with feeding difficulties(21). This study was conducted in Türkiye among children with drug resistant epilepsy. Hemamalini et al in India also found that feeding problems were present in 50% of children with epilepsy that were at high risk of malnutrition(6). We also found that participants with ongoing seizures were more likely to have acute malnutrition. Ongoing seizures indicate a form of uncontrolled epilepsy given that this was a population of children receiving treatment. Recurrent seizures may be associated with increased risk of hospitalisation and reduced energy intake. Furthermore, children with recurrent seizure activity have increased energy requirement which if not met may lead to malnutrition. There have also been studies that showed a two-way relationship between malnutrition and epilepsy revealing that malnutrition may reduce the seizure threshold as well as predispose to infections that provoke seizures (4, 6, 8).

School attendance offered reduced likelihood of acute malnutrition. This could be attributed to the fact that those attending school are more likely to be in better health compared to those that fail to attend school who are likely to have severe disease or disability. Additionally, school health programmes may offer children additional nutritious feeds while at school that improve the nutritional status of children. (22, 23). Although during the study period, most children were at home due to school closures resulting from the pandemic, good nutritional habits may have continued to prevail while at home. Social factors may also contribute to this finding because poor families that fail to send their children to school may also have low purchasing power for food. Rural residence and low level of education were two interesting factors that were associated with reduced odds of acute malnutrition. While some studies may find that rural areas have a bigger burden of malnutrition recent urbanisation and migration of people has deprived them of their food producing role and turned them into consumers that acquire food commercially. Caregivers with low education are more likely to reside in rural arears and take up subsistence farming that would increase the availability of food. Moreover, these caregivers are also more likely to be present at home and offer more care to the children. Our finding is similar to data reported by the Uganda Bureau of Statistics (UBOS) which found that children of caregivers with higher levels of education were more likely to be wasted even though they perform better on other growth parameters (24).

The strengths of our study include the fact that it was carried out at a referral hospital receiving patients from different areas of the country and with different types of epilepsy. Additionally, it included children with disabilities who are often excluded in other studies. Therefore, the study population was diverse, and the findings may be generalized to other children with epilepsy in a referral hospital setting in LMICs. One of our study limitations is that this study was carried out during the Covid-19 pandemic which could have influenced the feeding habits of families as well as socio economic effects. This could have given a different finding for the study compared to pre and post pandemic season. Secondly, the design of the study did not allow for observation of feeding practices and social economic status in the homes. However, clear and concise questions were asked to get the most accurate responses from the caregivers.

## Conclusion

This study demonstrated that the prevalence of acute malnutrition among children with epilepsy is high. Children with feeding difficulties, gross motor disability, and history of ongoing seizures in the last 6 months are more likely to have acute malnutrition. However, children residing in rural areas, those attending school and caregiver giver level of education of primary school or none were less likely to have acute malnutrition. There is thus need for targeted interventions aimed at improving nutrition status of children with epilepsy during their routine hospital care.

## Figures and Tables

**Table 1:** Demographic characteristics of participants and their caregivers

Characteristics (n=280)	Frequency(n)	Percentage%

**Age (years)**		
	
<5	162	57.9
	
≥ 5	118	42.1

**Sex**		
	
Male	187	66.8
	
Female	93	33.2

**Birth Order**		
	
1	105	37.7
	
2–3	112	40.3
	
≥4	61	22.0

**Residency**		
	
Urban	197	70.6
	
Rural	82	29.4

**Attending school**		
	
No	91	32.5
	
Yes	98	35.0
	
Not school going age	91	32.5

**HIV Status**		
	
Positive	6	2.1
	
Negative	274	97.9

**Birth order**		
	
1	105	37.8
	
2–3	112	40.3
	
≥4	61	22.0

**Caregiver**		
	
Parent	245	88.1
	
Relative	33	11.8
	
Other -orphanage, church pastor	2	0.7

**Level of education of the caregiver**		
	
Tertiary	62	22.3
	
Secondary	140	50.4
	
Primary/none	76	27.3

**Family monthly income (US Dollars)**		
	
≥ 200	43	16.9
	
100– <200	66	25.9
	
30– < 100	117	45.9
	
< 30	29	11.4

**Table 2:** Clinical characteristics of participants

Characteristic		Frequency (n)	Percentage (%)

**Duration of epilepsy**			
	
< 1 year		42	15.0
	
1–5 years		191	68.2
	
>5 years		47	16.8

**Age at onset**			
	
< 1 year		22	7.9
	
1 –5 years		140	50.0
	
>5 years		118	42.1

**Type of seizures**			
	
Generalized		161	57.5
	
Focal		119	42.5

**Seizure frequency**			
	
occasional*		134	47.9
	
Daily		96	34.3
	
Weekly		22	7.9
	
Monthly		28	10.0

**Anti-Epileptic drugs**			
	
None		11	4.0
	
Monotherapy		179	63.9
	
Polytherapy		90	32.1

**Use of Herbs**			
	
Yes		123	44.9
	
No		156	55.9

**Cormorbidities**			
	
Cerebral Palsy	Yes	103	36.9
	
Autism	Yes	3	1.1
	
Intellectual disability	Yes	7	2.5
	
Attention deficit	Yes	10	3.6

**Number of hospitalizations in the last year**			
	
< 2		169	60.8
	
>2		109	39.2

**Haemoglobin level**			
	
>/= 11.5g/dl)		157	56.8
	
<11.5g/dl		114	43.2

**Mean Cell Volume**			
	
>/= 75 to 86 fL		121	44.6
	
<75fl		150	55.4

**Table 3:** Multivariable analysis of factors associated with acute malnutrition among children with epilepsy.

			Acute Malnutrition			
Factor		Total	Yes (%)	No (%)	aOR (95% CI)	P Value
**Age(years)**	< 5	162	29	133	1.0	-
	≥ 5	118	15	103	0.70 (0.17–2.87)	0.62
**Sex**	Male	187	29 (65.9)	158 (66.9)	1.0	-
	Female	93	15 (34.1)	78 (33.1)	0.78 (0.29–1.99)	0.60
**Residency**	Urban	197	35 (81.4)	162 (68.6)	1.0	
	Rural	82	8 (18.6)	74 (31.4)	0.5(0.248–0.994)	0.048
**Attending School**	No	91	27 (61.4)	64 (27.1)	1.0	-
	Yes	98	3 (6.8)	95 (40.3)	0.12(0.03–0.41)	0.001
	Na	91	14 (31.8)	77 (32.6)	0.52(0.25–1.07)	0.076
**Gross Motor impairment**	No	180	8 (18.2)	172 (72.9)	1.0	-
	Yes	99	36 (81.8)	63 (26.7)	8.33 (2.27–34.5)	0.002
**Age at onset**	< 1 year	139	36 (81.8)	103 (43.6)	1.0	-
	1 year to 5 years	112	7 (15.9)	105 (44.5)	0.76 (0.21–2.72)	0.68
	>5 years	29	1 (2.3)	28 (11.9)	0.19 (0.01–3.20)	0.273
**Caregiver level of education**	Tertiary	62	12 (27.9)	50 (21.3)	1.0	-
	Secondary	140	22 (51.2)	118 (50.2)	0.83 (0.43–1.59)	0.574
	Primary/none	76	9 (20.9)	67 (28.5)	0.4(0.187–0.854)	0.018
**Food Insecurity**	No	216	28 (63.6)	188 (79.7)	1.0	-
	Yes	63	16 (36.4)	47 (19.9)	2.13 (0.77–5.92)	0.141
**Mean cell Volume**	Normal	121	14 (35.0)	107 (46.3)	1.0	
	Low	150	26 (65.0)	124 (53.7)	1.71 (0.96–3.02)	0.067
**Mother/guardian prepare food**	No	23	1 (2.3)	22 (9.3)	1.0	
	Yes	257	43 (97.7)	214 (90.7)	1.28 (0.16–28.1)	0.387
**Difficulty in feeding**	No	198	15 (34.1)	183 (77.5)	1.0	-
	Yes	81	29 (65.9)	52 (22.0)	3.19 (1.23–8.54)	0.018
**Hospital admission in last 1 year**	No	169	23 (52.3)	(61.9)	1.0	-
	Yes	109	21 (47.7)	88 (37.3)	0.98 (0.36–2.39)	0.882
**Seizures in last 6 months**	No	66	3 (6.8)	63 (26.7)	1.0	-
	Yes	214	41 (93.2)	173 (73.3)	5.65 (1.34–33.9)	0.032
**Stigma**	No	210	27 (62.8)	183 (77.2)	1.0	-
	Yes	70	16 (37.2)	54 (22.8)	1.3(0.693– 2.423)	0.417

## Data Availability

The original data set will be availed by the corresponding author upon reasonable request.

## Abbreviations

AEDs: Anti–Epileptic Drugs
CBC: Complete blood count
CT: computer tomography scan
CWE: Children/child living with epilepsy
EEG: Electro–encephalogram
GABA: Gamma–Aminobutyric Acid
HIV: Human Immuno–deficiency Virus
ILAE: International league against epilepsy
MAM: Moderate Acute Malnutrition
MRI: Magnetic resonance imaging
MUAC: Mid–Upper Arm Circumference
PI: Principal Investigator
PNC: Paediatric Neurology Clinic
PWEs: People Living With Epilepsy
SAM: Severe Acute Malnutrition
UDHS: Uganda Demographic Health Survey
WHO: World Health Organization
